# Purulent Pericardial Abscess Caused by Escherichia coli in an Immunocompromised Patient Presenting With Mixed Septic and Cardiogenic Shock

**DOI:** 10.7759/cureus.99534

**Published:** 2025-12-18

**Authors:** António L Pessoa, Ana Teixeira Reis, Ana Santos Costa, Miguel Rodrigues, Carolina de Almeida Robalo

**Affiliations:** 1 Internal Medicine, Centro Hospitalar de Setúbal, E.P.E., Setúbal, PRT

**Keywords:** immuno-compromised, mm: multiple myeloma, obstructive cardiogenic shock, purulent pericarditis, refractory septic shock

## Abstract

Purulent pericarditis and pericardial abscess are rare yet life-threatening complications of bacterial infection, particularly among immunocompromised individuals. We report the case of a 76-year-old woman with multiple myeloma undergoing chemotherapy who presented with lumbar pain, fever, hypotension, and acute kidney injury. Imaging revealed a large pericardial abscess with a distinct air-fluid level. Despite broad-spectrum antibiotics, fluid resuscitation, and high-dose vasopressor therapy, she progressed to mixed septic and cardiogenic shock. Urgent pericardiectomy drained purulent material that later cultured *Escherichia coli*, consistent with blood and urine culture results. Her clinical course was complicated by mediastinitis but improved with targeted antimicrobial therapy. She was discharged after 38 days. This case highlights the diagnostic challenges and the critical role of early imaging and prompt surgical intervention in purulent pericarditis, especially in immunocompromised hosts.

## Introduction

Purulent pericarditis is an uncommon but severe form of pericardial disease, accounting for less than 1% of all cases in the antibiotic era [1,2]. It is most frequently caused by Gram-positive organisms, whereas Gram-negative pathogens such as *Escherichia coli *are rare but increasingly recognized in immunocompromised individuals or in the setting of bacteremia [3,4,5]. Clinical manifestations are often nonspecific, and delayed diagnosis carries a high risk of morbidity and mortality. Early imaging with CT and echocardiography plays a critical role in establishing an accurate diagnosis and guiding treatment [6,7].

## Case presentation

A 76-year-old woman with IgG-λ multiple myeloma (Revised International Staging System Stage III (R-ISS III)), undergoing chemotherapy, presented with a 24-hour history of lumbar pain, fever, asthenia, and hypotension. Her medical history included chronic kidney disease (Kidney Disease: Improving Global Outcomes (KDIGO) G4), hypertension, paroxysmal atrial fibrillation, and anxiety-depressive disorder. Ten days earlier, she had been evaluated for fever and hypotension and started empirically on ciprofloxacin.

Upon arrival to the emergency department, she was somnolent but arousable, with blood pressure of 71/47 mmHg, mean arterial pressure (MAP) 55 mmHg, heart rate of 96 bpm, respiratory rate of 25 breaths/min, temperature of 35.4°C, and oxygen saturation of 96% on room air. Physical examination revealed muffled heart sounds, marked peripheral mottling, lower-limb edema, and anuria.

Laboratory tests showed leukopenia, thrombocytopenia, normocytic anemia, elevated inflammatory markers, metabolic acidosis, and hyperlactatemia (Table 1).

**Table 1:** Laboratory findings on arrival. BUN: blood urea nitrogen; HCO₃⁻​: bicarbonate ion.

Parameters	Result​	Reference range​
Hemoglobin​	8.9 g/dL​	11.5-15 g/dL​
Hematocrit​	26.5 %​	36-46 %​
Leukocytes​	3800 μL​	4500-11400 μL​
Neutrophils​	90.8 %​	41-75 % ​
Lymphocytes​	4.7 %​	20-48 %​
Platelets​	73000 μL​	150000-350000 μL​
BUN​	132 mg/dL​	21-43 mg/dL​
Creatinine​	2.37 mg/dL​	0.6-1.1 mg/dL​
Troponin I​	346.3 pg/mL​	13.8-17.5 pg/mL​
C-reactive protein​	26.16 mg/dL​	< 0.5 mg/dL​
pH​	7.274​	7.35-7.45​
HCO₃⁻​	10.3 mEq/L​	22-26 mEq/L​
Lactate​	7.8 mmol/L​	0.5-2.0 mmol/L​

**Figure 1:**
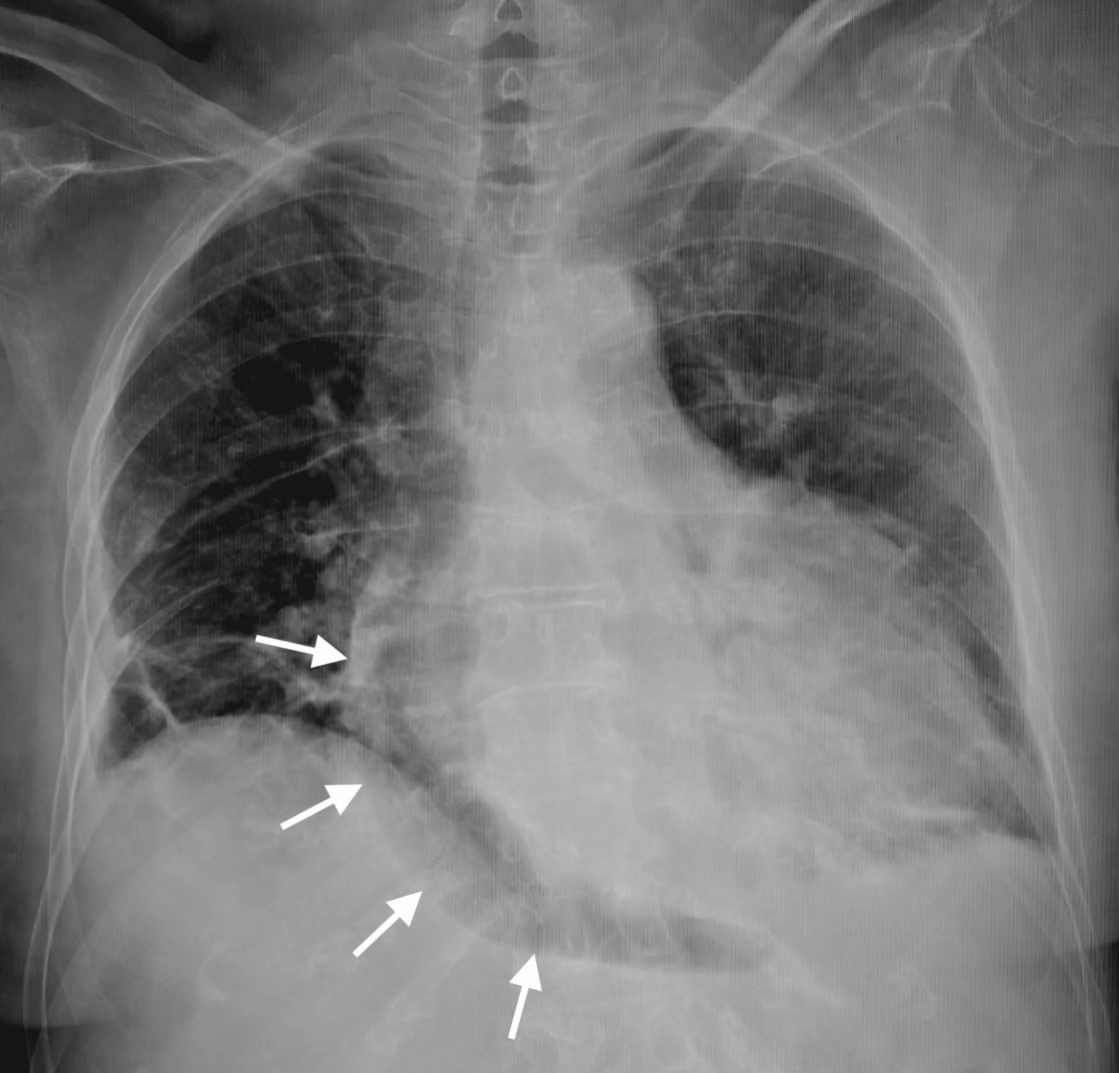
Anteroposterior (AP) chest radiograph. The image demonstrates a region of increased pericardial silhouette hypotransparency, an indirect radiographic sign suggestive of pericardial effusion.

Chest X-ray revealed a globular enlargement of the cardiac silhouette, an indirect radiographic sign suggestive of pericardial effusion (Figure 1).

**Figure 2:**
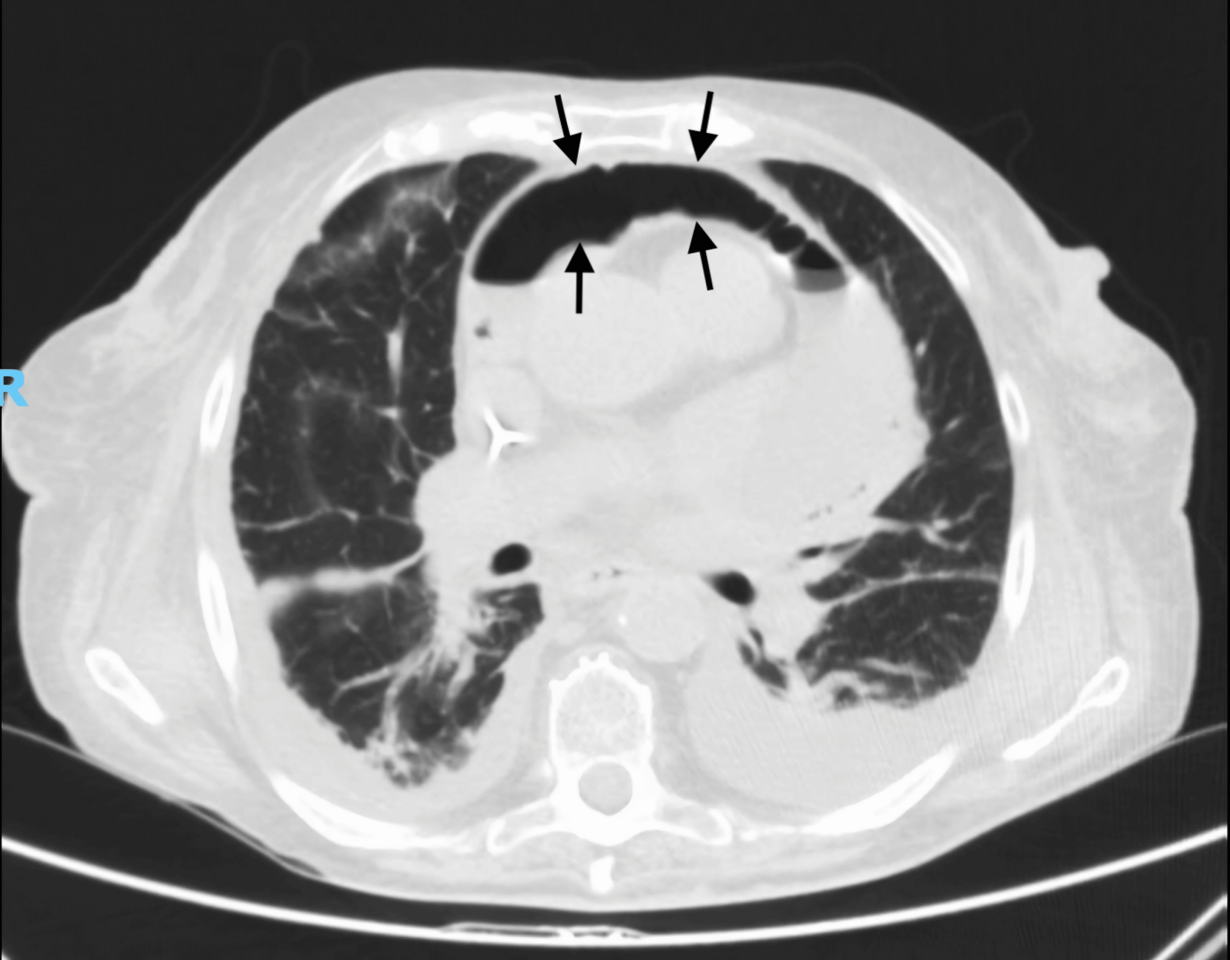
Axial contrast-enhanced CT of the chest in a pulmonary window setting (lung window). The image shows a large pericardial abscess with a distinct air–fluid level, causing partial compression of adjacent cardiac structures.

**Figure 3:**
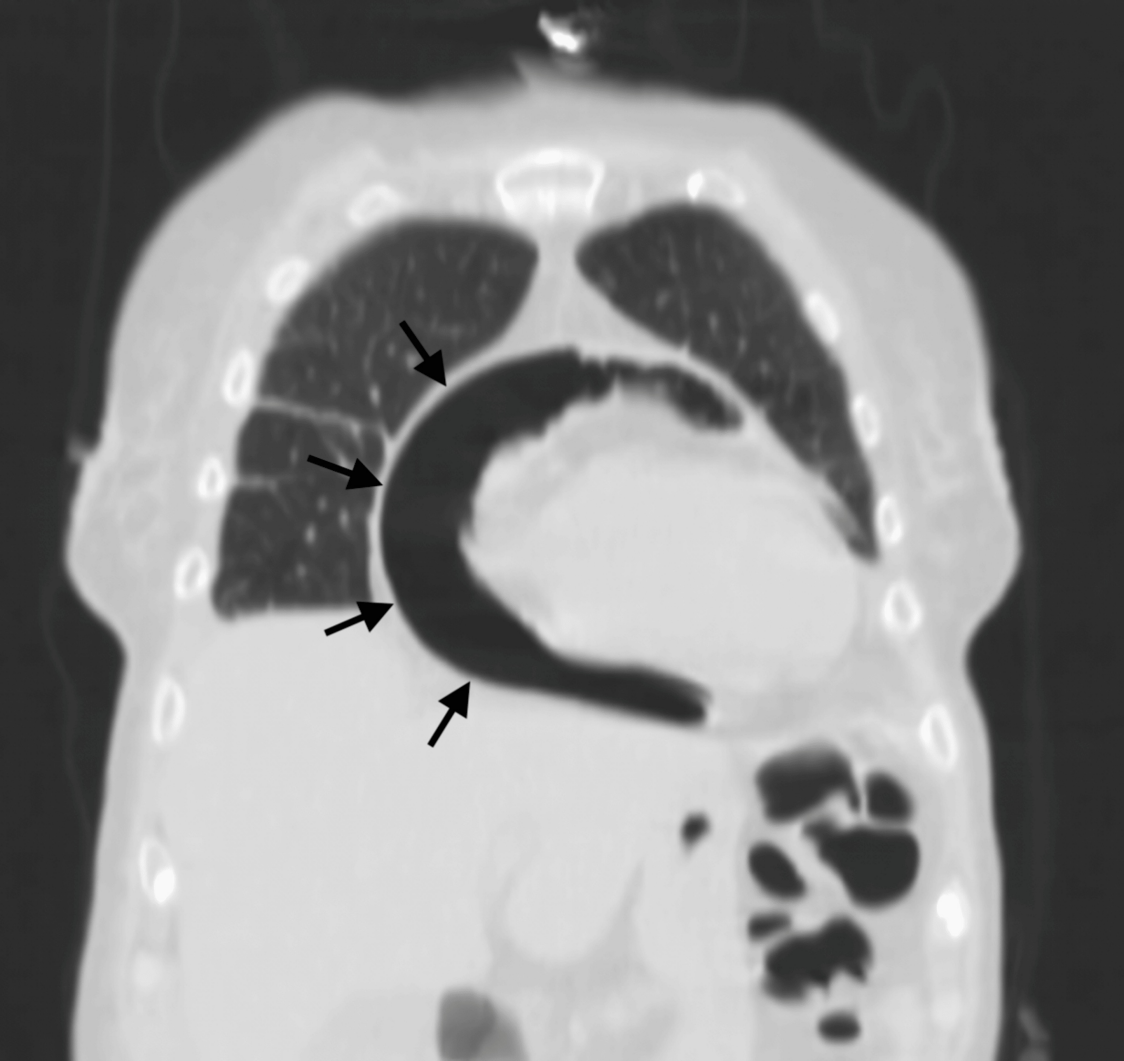
Coronal reconstruction of a contrast-enhanced CT scan in a pulmonary (lung) window. The image demonstrates an extensive purulent pericardial collection enveloping the heart, with a clear air–fluid interface.

CT imaging revealed a large pericardial abscess with an air-fluid level (Figures 2, 3). Transthoracic echocardiography demonstrated heterogeneous pericardial collections suggestive of purulent content and impending constrictive physiology. Despite broad-spectrum antibiotics and vasopressor support, the patient progressed to mixed septic and cardiogenic shock.

Given worsening hemodynamic instability, she underwent urgent pericardiectomy with drainage of copious purulent material. Cultures from pericardial fluid, blood, and urine yielded *E. coli* demonstrating resistance to ampicillin and trimethoprim-sulfamethoxazole and susceptibility to amoxicillin-clavulanate, cefuroxime, cefotaxime, and piperacillin-tazobactam, the latter of which was used for prolonged postoperative therapy due to mediastinitis. She recovered and was discharged after 38 days.

## Discussion

Purulent pericarditis and pericardial abscess carry high mortality if not promptly diagnosed and treated. Although Gram-positive organisms remain the most common etiologies, Gram-negative pathogens are increasingly recognized in immunocompromised or bacteremic patients. *Escherichia coli* is an especially uncommon cause, with only scattered case reports-typically in patients with hematologic malignancies, postoperative thoracic infections, or urinary-tract-related bacteremia [4,5,8,9-12]. Our patient shared several classical risk factors, including advanced multiple myeloma, chemotherapy-induced immunosuppression, and urinary infection as the likely portal of entry.

Symptoms often mimic septic shock of non-cardiac origin, contributing to significant diagnostic delay. Several authors highlight that early manifestations are nonspecific and that cardiac involvement may remain unrecognized until imaging is performed [3,13,14]. CT is particularly valuable for identifying pericardial air-fluid levels, loculated collections, or mediastinal involvement, findings strongly suggestive of purulent infection, while echocardiography provides essential information regarding hemodynamic compromise and evolving constrictive physiology [7,8,14]. In our patient, the presence of a pericardial abscess with a well-demarcated air-fluid interface mirrors radiologic findings described in a minority of previously reported *E. coli* cases [9,11-13].

Definitive management requires directed antimicrobial therapy and drainage of the infected pericardial space. Although pericardiocentesis may be adequate for simple effusions, several retrospective series have demonstrated higher recurrence rates and inadequate source control with percutaneous drainage alone in cases of bacterial pericarditis [6,10,15]. Surgical drainage or pericardiectomy is preferred in cases involving loculated, thickened, or adherent pericardium, as seen in our patient. Early surgical intervention, combined with targeted antimicrobial therapy, led to successful recovery despite complications. This outcome aligns with recent reports indicating that timely and aggressive drainage remains the strongest predictor of survival in purulent pericarditis, particularly in immunocompromised hosts [9,10,14].

## Conclusions

Pericardial abscess due to *E. coli* is exceedingly rare but should be considered in immunocompromised patients presenting with shock and sepsis. Early CT imaging, prompt antimicrobial therapy, and surgical drainage are critical for favorable outcomes. This case highlights the need for high clinical suspicion and early multidisciplinary management.
